# Lipid oxidation, sensory characteristics, and color of fresh pork sausage from immunologically castrated pigs stored frozen for up to 12 weeks

**DOI:** 10.1002/fsn3.297

**Published:** 2015-10-09

**Authors:** Katelyn A. Jones‐Hamlow, Marcos A. Tavárez, Aubrey L. Schroeder, Anna C. Dilger

**Affiliations:** ^1^ Department of Animal ScienceUniversity of Illinois1503 S. Maryland Dr.UrbanaIllinois61801; ^2^ Veterinary Medicine Research & DevelopmentZoetisKalamazooMichigan49007

**Keywords:** Boar taint, discoloration, frozen storage, immunological castration, pork, retail display

## Abstract

Two studies were conducted to evaluate the quality characteristics of fresh sausage manufactured from immunologically castrated (IC) pigs, an emerging technology in the pork industry. Study 1: Fresh sausage patties from ground Boston butts fabricated from PC (physically castrated) pigs fed 0.55% SID (standard illeal digestible) lysine, IC pigs fed 0.55% SID lysine, and IC pigs fed 0.65% SID lysine were made and not standardized to a similar content of fat content. Study 2: fat and lean trim obtained from IC and PC pigs was made into fresh sausage patties, targeting 25% lipid. Patties (1.25 cm) were placed on trays and assigned to 0, 4, or 12 weeks frozen storage and then, after frozen storage, placed in simulated retail display conditions for 5 days. Patties were evaluated for color stability, sensory and textural properties, and lipid oxidation. Data were analyzed as a one way ANOVA with repeated measures where appropriate. In both studies, sausage discolored with both increased time in frozen storage and with increased time in retail display (*P < *0.01). However, within each week of frozen storage, all treatments were equally discolored in both studies. Treatment did not affect the sensory characteristics or textural properties of fresh sausage in either study. Lipid oxidation did not differ by treatment in study 1. In study 2, lipid oxidation was reduced (*P *<* *0.05) in IC compared to PC by approximately 0.1 mg malonaldehyde/kg meat, but there were no differences within each storage week. Increasing frozen storage time increased lipid oxidation an off‐flavor scores in both studies (*P *<* *0.05). Overall, immunological castration had no detrimental effects on color stability, sensory characteristics, or textural properties of fresh pork sausage.

## Introduction

Improvest^®^ (Zoetis, Kalamazoo, MI) is immunological product for intact male pigs that stimulates the production of antibodies against gonadotropin‐releasing factor (GnRF), blocking its activity and causing temporary castration. It was approved for use in the United States in 2011 (FDA 2011). Improvest management delays castration, taking advantage of increased feed efficiency and lean gain of intact male pigs (Dunshea et al. 2001; Boler et al. 2011), without the negative sensory characteristics associated with boar taint (Font i Furnols et al. 2008, 2009). However, like any new technology used in the food industry, careful investigation of the quality and color stability of pork produced from Improvest‐managed pigs is needed to determine any potential differences that may limit the acceptance in the marketplace.

According to the National Pork Board (2009)AUTHOR: National Pork Board (2009) has not been included in the Reference List, please supply full publication details., 20% of pork is consumed as sausage products. Thus, the effect of immunological castration on the color stability and sensory characteristics of pork sausage must be characterized. Growth rate of immunologically castrated (IC) pigs is similar to boars until administration of the second of two Improvest injections. Therefore, IC pigs may have fatty acid profiled with increased concentrations of PUFA (polyunsaturated fatty acids) more similar to boars than PC (physically castrated) pigs (Mackay et al. 2013). Sausage with increased concentrations of PUFA is more susceptible to oxidative deterioration of lipids (Rhee et al. 1988; Leskanich et al. 1997) causing discoloration (Baron and Andersen 2002) and rancid off flavors (Kanner 1994). In emulsified products, increased PUFA causes products to be softer and less desirable (Bloukas and Paneras 1993). Therefore, the potential increased PUFA in meat from IC pigs may be detrimental to sausage color stability and palatability.

The objectives of the following studies were to evaluate the lipid oxidation and sensory characteristics of fresh sausage patties and color evaluation during retail display of frozen sausage patties from immunologically castrated and physically castrated pigs.

## Materials and Methods

Two studies were conducted to address two different scenarios of sausage manufacturing. The first study formulated sausages from whole, boneless Boston Butts (IMPS#406A). Immunologically castrated pigs have leaner carcasses (Boler et al. 2011). Therefore, by manufacturing sausage from whole, boneless Boston butts, the fat content of sausage in study 1 was reduced in IC compared to PC. It is typical in sausage manufacturing, however, to produce sausages with consistent fat contents. To achieve this, lean and fat trim are combined to achieve a targeted fat content. Thus, study 2 used fat and lean trim from IC and PC pigs to formulate sausage with a targeted lipid content of 25%.

Experimental procedures during the live phase of the following studies followed guidelines for the Care and Use of Agricultural Animals in Agricultural Research and Teaching (FASS 1999).

### Animal selection and raw material preparation study 1

Pigs used for this study were selected from a larger experiment in which approximately 1000 pigs of commercial breeding comparable to those used in industry were allotted to a wean‐to‐finish building. Treatments from the larger study included PC barrows fed 0.55% standard ileal digestible (SID) lysine and IC barrows fed 0.55% and 0.65% SID lysine for approximately 7 weeks prior to slaughter. Diets were formulated to meet or exceed National Research Council (NRC) requirements for PC barrows. As such, PC barrows were fed 0.55% SID lysine and one treatment of IC barrows were fed similarly. However, to maximize growth of IC barrows, more lysine is required (Boler et al. 2011), therefore, one treatment of IC barrows included 0.65% SID lysine. Physically castrated barrows were castrated at or before 7 days of age, while IC barrows received the first injection of Improvest^®^ at ~4 months of age (16 weeks) and the second injection at 20 weeks of age. Pigs were transported to a commercial facility and harvested under inspection at an approximate ending live weight of 120 kg (27 weeks of age). Carcasses from the two pigs closest to the pen mean weight were shipped to University of Illinois Meat Science Laboratory for this study. Therefore, in total, 42 carcasses representing 21 pens (*n* = 7 pens per treatment) were used in this study. Upon arrival, carcasses were fabricated according to specifications described by the National Association of Meat Purveyors (NAMP 2007). Boston Butts (NAMP #407) were obtained to be used in this experiment. Boston butts were paired by pen (2 pigs/pen), ground and allotted into a single 4.5 kg batch per pen. A total of 21 batches were prepared 8 days after slaughter.

### Animal selection and raw material preparation study 2

Five IC pigs were transported to and slaughtered under inspection at the University of Illinois Meat Science Laboratory. Similar to study 1, IC pigs received the first injection of Improvest at ~4 months of age (16 weeks) and the second Improvest injection 4 weeks later at 20 weeks of age. All IC pigs were slaughtered at 27 weeks of age, 7 weeks after the second injection. Five contemporary PC pigs, castrated at or before 7 days of age, from the same facility were slaughtered at the same time. All pigs, regardless of castration method, were fed similar diets. Fat and lean trim were obtained from entire carcasses and pooled by castrate type 1 day after slaughter and stored in vacuum bags until fabrication. Subsamples of trim were collected for proximate analysis (method below). Based on these results, fat and lean trim were weighed and allotted into five independent, 6.8 kg batches for each treatment, targeting 25% lipid. A total of 10 batches were prepared 8 days after slaughter.

### Sausage preparation

For the remainder of the methods, both studies were similar. To prepare fresh sausage patties, batches were mixed in a bowl chopper with 5.8 g/kg salt, 1.4 g/kg black pepper, and 3.1 g/kg dextrose and stuffed into 4 × 3 × 26 poly‐bag casings. Sausages were crust frozen and cut into 1.25 cm thick patties using a band saw. From each experimental unit, six sausage patties were designated for display with two patties randomly assigned to each of three frozen storage times and packaged on a single tray. Patties were placed on trays and packaged with PVC overwrap (oxygen transmission rate = 11,627.9 cc/m^2^/day; moisture vapor transmission rate = 170.5 gm/m^2^/day) and assigned to 3 frozen storage times: 0, 4 or 12 weeks. Samples were held at −20°C for duration of assigned storage time. Two additional patties were stored in boxes and held at −20°C for analysis of break strength and compression analysis.

### Proximate analysis

For raw proximate analysis, a subsample of sausage was obtained prior to stuffing into casings. The subsample was ground in a Cusinart Food Processor (Model DLC 5‐Tx; Cuisinart, Stamford, CT), and a 10‐g sample of homogenate was oven‐dried at 110°C for approximately 24 h to determine percent moisture. Lipid from the dried sample was extracted using an azeotropic mixture of warm chloroform:methanol (4:1) as described by Novakofski et al. (1989) to determine the extractable lipid.

### Evaluation of sausage patties in simulated retail display

For both studies, upon conclusion of frozen storage, sausage patties were displayed in packages under constant lights (883 lx) at 4°C for 5 day. On days 1, 3, and 5, an experienced, five‐person panel evaluated patties for discoloration and overall color. Evaluations were conducted at the same time for each display day. Trays were identified with unique numbers, therefore, panelists were blinded to treatment. Panelists received standards to evaluate metmyoglobin formation for discoloration and National Pork Producers Council (NPPC 1999) color guide to evaluate overall color similar to the methods described in Boler et al. (2009). Discoloration was evaluated, using a 10 cm line scale (reference marks at 0.0, 2.5, 5.5, 7.5, and 10.0 cm), where every 1 cm represented 10% discoloration of both patties on the tray similar to methods described by Holmer et al. (2009). Panelists also evaluated sausage patties for overall color, using a 10 cm unstructured line scale where 0 represented a color score 1 (very pale), 5 represented a color score 3.5 (average), and 10 represented a color score 6 (very dark) according to NPPC color standards (NPPC 1999a). Values recorded were then converted into the NPPC 6‐point color scale using the equation: 0.5 (color score) + 1.

### Sensory characteristics of fresh sausage patties

Upon conclusion of simulated retail display (5 day), sensory evaluation was conducted on one patty per tray using a trained panel composed of individuals from the University of Illinois Meat Science Laboratory. For study 1, 21 sausage patties (*n* = 7/treatment) were evaluated in four sessions (three sessions with six samples and one session with three samples). The first two sessions occurred on display day 5, and the remaining sessions occurred the following day. For study 2, 10 sausage patties (*n* = 5/treatment) were evaluated during two sessions (one session with six samples and one session with four samples). Both sessions occurred on the same day. For both studies, patties were allotted into panels such that each treatment was balanced in each panel and every panelist consumed a subsample of each patty.

Panels consisted of six members who were trained according to the guidelines of the American Meat Science Association (AMSA 1995). Panelists were separated by booths and evaluated the patties under red lighting. Water and unsalted crackers were provided between each sample. Panelists were instructed to remain consistent with either swallowing or expectorating samples.

Patties were cooked in a 176°C oven for 14 min to reach a target internal temperature of 70°C. Panelists evaluated sausage patties for juiciness, mouthfeel, and off flavor on a 15 cm unstructured line scale anchored at the center and both ends where 0 cm represented extremely crumbly, very dry, and no off flavor, and 15 cm represented extremely chewy/rubbery, very juicy, and intense off flavor.

### Thiobarbituric acid relative substances (TBARS)

Upon conclusion of simulated retail display, one patty per tray was homogenized in a Cusinart Food Processor (Model DLC 5‐Tx; Cuisinart) for TBARS analysis, using a modified version of the procedure described by Leick et al. (2010). Briefly, 5 g of the homogenate was blended with butylated hydroxytoluene (BHT) and trichloroacetic acid and then filtered. Filtrate was added to 0.02 mol/L thiobarbutic acid. A standard curve (0, 1.25, 2.5, 5.0, and 7.5 mg [malonaldehyde] MDA/mL) was prepared. Samples and standards were incubated in a dark cabinet for 18 h at 24°C. After incubation, samples were placed in a 96‐well plate and absorbance read at 530 nm in a plate reader (Synergy HT Multi‐Mode Microplate Reader; Bio‐Tek, Winooski, VT) to determine MDA content. Samples were compared to a standard curve (0–22.5 mg MDA/mL) and TBARS were expressed as mg MDA/kg meat.

### Textural analysis

Upon conclusion of all frozen storage times, sausage patties, stored frozen for 12 weeks, were cooked similarly to those for sensory panels and weighed before and after cooking to determine cook loss. After cooking, patties were allowed to cool at room temperature for 1 h. Break strength (kg force) was determined using methods previously described by Bess et al. (2013).

An additional patty was cooked in a similar fashion and used to determine hardness, fracturability, adhesiveness, springiness, cohesiveness, gumminess, chewiness, and resilience according to the methods of Bourne et al. (1978). Four 2.54 cm cores were collected from each sample and compressed on the Texture Analyzer TA.HD Plus (Texture Corp., Scarsdale, NY; Stable Microsystems, Godalming, UK). A 5.08 cm diameter plate compressed each core into two consecutive cycles to 75% of the sample original height in 2 sec intervals between cycles. The cross‐head moved at a constant speed of 5 mm/sec. A force‐time curve was plotted and the peak force of the first compression was used to determine parameters previously mentioned. The values of the four cores were averaged and reported as hardness, fracturability, adhesiveness, springiness, cohesiveness, gumminess, chewiness, and resilience of each patty.

### Statistical analysis

For both studies, the batch served as the experimental unit for all traits. In study 1, there were seven batches (1 per pen of pigs) for each treatment for a total of 21 batches. For study 2, there were five batches for each treatment for a total of 10 batches. Data were analyzed using the MIXED procedure of SAS (SAS Institute Inc., Cary, NC). Color during display, sensory, and TBARS data were analyzed as repeated measures over time, using an unstructured (UN) covariate matrix based upon goodness‐fit‐analysis, using Akaike's information criterion to minimize variance. The statistical model for color during display included the fixed effects of treatment, day of display, frozen storage time, and all interactions. The statistical model for sensory and TBARS included the fixed effects of treatment, frozen storage time, and their interactions. Textural properties and proximate analysis were analyzed as a one way analysis of variance (ANOVA) with the fixed effect of treatment. Assumptions of ANOVA were tested with Levene's test and Brown and Forsythe for homogeneity of variances. Normality of residuals was tested, using the Univariate procedure of SAS. Means were separated using the PDIFF option, employing a Tukey's adjustment for multiple comparisons. Differences were deemed significant at *P *≤* *0.05.

## Results and Discussion

### Effect of immunological castration on characteristics of fresh sausages

#### Proximate composition

For study 1, proximate composition (Table 1) of fresh sausage was affected by treatment. Sausage from IC pigs fed on 0.65% SID lysine had 5.57 percentage units more (*P *<* *0.05) moisture and 7.23 units less (*P *<* *0.05) fat compared with sausage from PC pigs. Sausages from IC pigs fed 0.55% SID lysine were intermediate to but not different (*P *>* *0.05) from the other two treatments. Given that these sausages were manufactured from whole Boston butt shoulders of IC and PC pigs, it is not surprising that the sausages of IC pigs had reduced fat compared to those of PC pigs. Several authors have noted reductions in carcass fatness in IC pigs (Dunshea et al. 2001; Boler et al. 2011; Lowe et al. 2014a,b). However, sausages would typically be standardized to a given content of fat as in study 2. In those sausages, there were no differences (*P *=* *0.12) between treatments for the fat content of sausages (Table 1).

**Table 1:** Characteristics of fresh sausages from physical castrates (PC) and immunological castrates (IC) fed 0.55% and 0.65% standard ileal digestible lysine (study 1) and fresh sausages formulated with equal amounts of fat from PC and IC pigs (study 2)

Item	Study 1					Study 2			
	PC‐0.55	IC‐0.55	IC‐0.65	SEM	*P*‐Value	PC	IC	SEM	*P*‐Value
Moisture, %	62.71^a^	65.96^ab^	68.28^b^	1.13	<0.01	58.30	57.60	0.002	0.05
Lipid, %	21.66^a^	17.13^ab^	14.43^b^	1.43	<0.01	24.60	25.20	0.002	0.12
Discoloration, %^a^	7.38	6.65	7.00	0.71	0.91	13.77	12.85	0.03	0.05
Color^a^	3.62^a^	3.81^b^	3.66^a^	0.04	<0.01	3.42	3.75	0.05	<0.01
Juiciness	8.40	8.36	8.46	0.12	0.13	8.60	8.33	0.19	0.35
Mouthfeel	8.65	8.40	8.46	0.11	0.18	7.94	8.11	0.12	0.33
Off‐flavor	1.25	1.24	1.67	0.20	0.28	–	–	–	–
Thiobarbituric acid relative substances, mg malonaldehyde/kg meat	0.23	0.18	0.18	0.03	0.45	0.47	0.36	0.03	0.05
Break strength, kg	4.87	6.50	6.10	0.82	0.49	5.397	4.989	0.320	0.39
Hardness, kg	13.34	12.94	15.05	8.01	0.16	18.38	20.17	14.04	0.40
Fracturability, kg	12.11	12.14	14.48	12.14	0.31	18.38	20.17	14.04	0.40
Adhesiveness	−0.01	−0.01	−0.01	0.002	0.73	−0.01	−0.03	0.01	0.19
Springiness	0.62	0.66	0.69	0.03	0.34	0.603	0.617	0.019	0.62
Cohesiveness	0.28	0.28	0.28	0.01	0.99	0.256	0.266	0.007	0.33
Gumminess, kg	3.51	3.71	4.61	4.24	0.18	4.71	5.44	5.14	0.35
Chewiness, kg	2.34	2.46	2.83	2.68	0.40	2.86	3.41	3.99	0.35
Resilience	0.08	0.80	0.82	0.00	0.89	0.063	0.066	0.001	0.09

Means within row within study lacking common superscripts are different (*P* < 0.05).

a Discoloration and color were determined by a panel evaluation using references.

#### Color

In both studies, the interaction of treatment (PC, IC) with day of display, frozen storage time, or the interaction of all three effects was not significant (*P *≥* *0.09) for either color or discoloration (Table 1). In study 1, when fat differences between IC and PC pigs were not controlled, treatment did not alter discoloration (*P = *0.91). However, in study 2, when all sausages were standardized to a similar fat content, discoloration was increased (*P = *0.05) marginally, by less than a percentage unit, in sausages from PC pigs compared with IC pigs.

It was anticipated that sausage from IC pigs might exhibit increased discoloration due to the potential increased PUFA in IC pigs. Previous studies have indicated that increased PUFA results in increased lipid oxidation and discoloration (Faustman et al. 2010). Discoloration, however, was not increased in IC pigs. This may be attributed to the length of time after second Improvest dose in this study. Increased PUFA have been noted in IC pigs slaughtered at 3–5 weeks after the second injection compared to PC pigs (Mackay et al. 2013). But at 6–8 weeks after the second injection, PUFA content was similar between IC and PC pigs (Tavárez et al. 2014b). Pigs in this study were slaughtered 7 weeks after the second injection, and therefore, may not have differed in PUFA concentrations compared with PC pigs. Furthermore, increased discoloration of sausage from PC pigs by approximately one percentage units, while statistically significant, is likely not great enough in magnitude to alter consumer purchasing decisions.

In study 1, when fat differences between IC and PC pigs were not controlled, treatment (*P < *0.01) did affect overall color. When averaged across all frozen storage points, sausage from IC barrows fed 0.55% lysine had marginally darker color compared with PC pigs and IC pigs fed 0.65% lysine. In study 2, when all sausages were standardized to a similar fat content, sausage from IC pigs was darker (*P < *0.01) than sausage from PC pigs. In both studies, the magnitude of differences was very small and within a range which consumers would consider normal colored. When consumers were asked to evaluate pale, soft, and exudative, normal, and dark, firm, and dry pork, they rated normal colored pork from 2.6 to 3.6 on a 5‐point scale (Brewer et al. 1998). This would correspond to a 3.1–4.3 on the NPPC 6‐point scale resulting in color ratings similar to our study. These minimal differences in overall color between sausages of IC and PC pigs are similar to results several other studies of loin color. When comparing IC to PC pigs, some authors have reported darker loins (Jones‐Hamlow et al. 2015), others report lighter loins (Tavárez et al. 2014a), and others detected no differences (Boler et al. 2014; Lowe et al. 2014b). Even when differences were detected, they were minimal in magnitude, similar to the present study in sausages. Therefore, color differences between products of IC and PC pigs should not be distinguishable to the consumer.

#### Sensory

**Figure 1:**
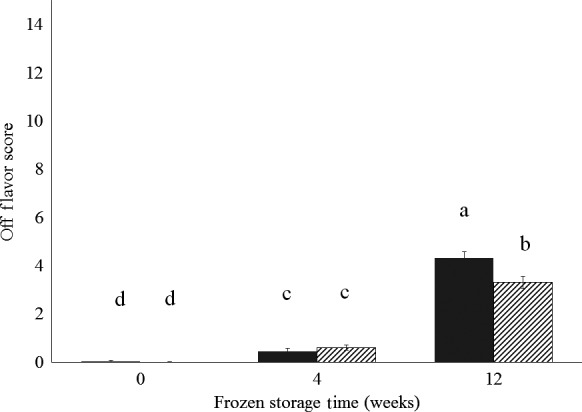
Effect of immunological castration and frozen storage on the off flavor of pork sausage standardized to a similar fat content from immunologically castrated (hashed bars) and physically castrated (solid bars) pigs. Columns represent least square means with standard error bars. Columns lacking common superscripts are different (*P* < 0.05).

In either study, juiciness (*P *≥* *0.13) or mouth‐feel (*P *≥* *0.18) did not differ between treatments (Table 1). In study 1, off flavor was also not different (*P = *0.28) between treatments. For study 2, however, there was an interaction between treatment and frozen storage time for off flavor (*P < *0.01). Off flavor did not differ between PC and IC pigs after 0 and 4 weeks of frozen storage, but after 12 weeks of frozen storage, off flavor was increased in sausage from PC pigs compared with that from IC pigs (Fig. 1).

#### Lipid oxidation

For both studies, there was no interaction between treatment and frozen storage time (*P > *0.50). In study 1, when fat differences between IC and PC pigs was not controlled, lipid oxidation (Table 1) was not affected by treatment (*P = *0.34). However, in study 2, when all sausages were standardized to a similar fat content, lipid oxidation was increased (*P = *0.05) in sausage from PC pigs (0.47 mg MDA/kg of meat) when compared to IC pigs (0.22 mg MDA/kg of meat). An increase in TBARS was predicted in IC pigs. Previous work has demonstrated an increase in PUFA in IC pigs compared with PC pigs (Asmus et al. 2014), which would increase TBARS. However, others have demonstrated that at 6–8 weeks after the second injection, PUFA content was similar between IC and PC pigs (Tavárez et al. 2014b). Thus, it is possible that in this study where IC pigs were slaughtered 7 weeks after the second injection, PUFA concentrations did not differ between IC and PC pigs. Therefore, lipid oxidation was not increased in IC pigs.

#### Texture

For both study 1 and 2, break strength of sausage and textural properties did not differ (*P *>* *0.09) between IC and PC pigs (Tables 2 and 3). The lack of differences in the textural properties of IC and PC pigs could be due to the differences in fat content and PUFA concentration not being great enough to detect differences. While fat has a major influence on the binding properties, tenderness, juiciness, mouthfeel, and overall appearance of emulsified meat products (Sofos and Allen 1977; Hand et al. 1987), it is possible that in this study, the differences in fat content and PUFA concentrations were not great enough to detect differences. Baer and Dilger (2014) also observed no differences in fresh sausage which had a differing iodine value of 4 g/100 g. Thus, it is possible that the PUFA concentrations in fresh sausage from these two studies were not great enough to note differences in break strength.

**Table 2:** Characteristics of fresh sausages stored frozen for 0, 4, or 12 weeks from physical castrates and immunological castrates but not standardized to a common fat content (study 1) and fresh sausages formulated with equal amounts of fat from physically castrated and immunologically castrated pigs (study 2)

Item	Study 1					Study 2				
	Wk 0	Wk 4	Wk 12	SEM	*P*‐Value	Wk 0	Wk 4	Wk 12	SEM	*P*‐Value
Juiciness	8.24^a^	8.63^b^	8.60^b^	0.07	<0.01	8.37	8.32	8.70	0.15	0.22
Mouthfeel	8.56^ab^	8.80^a^	8.32^b^	0.07	<0.01	7.96^ab^	7.78^b^	8.33^a^	0.10	0.05
Off‐flavor	0.15^a^	0.99^b^	2.83^c^	0.16	<0.01	–	–	–	–	–
Thiobarbituric acid relative substances, mg malonaldehyde/kg meat	0.11^a^	0.18^b^	0.30^c^	0.01	<0.01	0.22^a^	0.42^ab^	0.60^b^	0.02	<0.01

Means within row within study lacking common superscripts are different (*P* < 0.05).

**Table 3:** Characteristics of fresh sausages stored frozen for 0, 4 or 12 weeks from physical castrates and immunological castrates but not standardized to a common fat content (study 1) and fresh sausages formulated with equal amounts of fat from physically castrated and immunologically castrated pigs (study 2)

Item	Week 0			Week 4			Week 12			SEM^a^
	d1	d3	d5	d1	d3	d5	d1	d3	d5	
Study 1^b^										
Discoloration, %^c^	0.0^f^	1.9^e^	4.3^d^	0.7^ef^	6.9^cd^	9.1^bc^	7.9^bcd^	12.8^b^	19.3^a^	1.10
Color^c^	3.8^abc^	3.6^bc^	4.0^a^	3.8^ab^	3.9^a^	3.9^a^	3.6^cd^	3.6^cd^	3.4^d^	0.06
Study 2^d^										
Discoloration, %^c^	0.0^d^	0.2^d^	0.6^d^	0.7^d^	2.4^d^	4.9^c^	15.1^b^	49.2^a^	46.7^a^	0.60
Color^c^	3.5^bc^	3.3^c^	3.5^bc^	3.9^a^	3.8^ab^	3.4^c^	3.4^c^	3.7^ab^	3.7^ab^	0.07

Means within row within study lacking common superscripts are different (*P* < 0.05).

a Greatest SEM of treatments was reported.

b Sausages not standardized to a common fat content.

c Discoloration and color were determined by a panel evaluation using references.

d Sausages formulated with equal amounts of fat.

### Effects of display time and frozen storage time on characteristics of fresh sausages

In both studies, there was an interaction between display day and frozen storage time (*P < *0.01) for both color and discoloration. As frozen storage time increased, the rate or extent of discoloration during display increased. In study 1 (Table 3), discoloration of sausages stored for 12 weeks was greater (*P < *0.05) than that of sausages stored for 0 and 4 weeks at each display day. Conversely, discoloration on display day 1 was similar between sausages frozen for 0 and 4 weeks, but at 3 and 5 days of display, discoloration was greater (*P < *0.05) in sausages stored frozen for 4 weeks than those never frozen. In study 2, there were no differences in discoloration among sausages stored frozen for 0 or 4 weeks and displayed for 1 or 3 days. However, discoloration was greater (*P < *0.05) in sausages stored frozen for 12 weeks at all display days than those stored frozen for 0 and 4 weeks. These differences in discoloration were expected because metmyoglobin formation increases with storage time (Ledward and Macfarlane 1971; Wanous et al. 1989) due to the oxidation of iron in the heme ring (Fox 1966). Results of color with display were more variable. In study 1 (Table 3), sausages stored frozen for 12 weeks were darker (*P < *0.05) at each display day than those stored frozen for 0 and 4 weeks. However, in study 2, sausages stored frozen for 12 weeks were lighter (*P < *0.05) than those stored frozen for 0 and 4 weeks on display days 3 and 5. These changes in color, though, were <0.5 units, and therefore, are likely not to alter consumer purchasing decisions.

#### Sensory

In general, the alterations in sensory properties with frozen storage were minor. In study 1, where sausages were not standardized to a similar fat content between treatments, sausage patties stored for 0 weeks were drier (*P < *0.05) than those stored for 0 or 4 weeks (Table 2), but juiciness did not differ with frozen storage time in study 2. In study 1, sausages stored frozen for 4 weeks were less (*P < *0.05) crumbly than those stored frozen for 12 weeks. However, in study 2, sausages stored for 4 weeks were more crumbly (*P < *0.05) than those stored for 12 weeks. In both studies, increasing time in frozen storage increased (*P < *0.05) off‐flavor (Table 2, Fig. 1).

#### Lipid oxidation

In both studies, as frozen storage time increased (*P < *0.01), lipid oxidation also increased (Table 2). However, all TBARS for both studies were below the maximum detectable threshold of 1.0 *μ*g of MDA/kg of meat for all weeks of frozen storage times (Tarladgis et al. 1960). As indicated by off flavor scores as well, excessive lipid oxidation that would result in decreased consumer acceptance did not occur in any treatment in these studies.

## Conclusion

With 20% of pork consumed as sausage (Pork Checkoff 2009), it is imperative to understand the effects a new technology such as immunological castration may have on the color stability, color stability and sensory properties of fresh sausage. Results from both studies suggest that immunological castration does have a detrimental effect on color stability of fresh sausage. Immunological castration also did not negatively affect the lipid oxidation or sensory characteristics of fresh sausage in either study. Therefore, pork from immunologically castrated pigs can be used similarly to that of physically castrated pigs without detrimental effects on the quality of fresh pork sausage.

## Conflict of Interest

None declared.
